# Unveiling the Immunomodulatory Characteristics of *Haemonchus contortus* Ephrin Domain Containing Protein in the Parasite–Host Interactions

**DOI:** 10.3390/ani10112137

**Published:** 2020-11-17

**Authors:** Kalibixiati Aimulajiang, Zhaohai Wen, Xiaowei Tian, Shakeel Ahmed Lakho, Yang Zhang, Muhammad Ali-ul-Husnain Naqvi, Meng Liang, Xiaokai Song, Lixin Xu, Xiangrui Li, Ruofeng Yan

**Affiliations:** MOE Joint International Research Laboratory of Animal Health and Food Safety, College of Veterinary Medicine, Nanjing Agricultural University, Nanjing 210095, China; 2017207022@njau.edu.cn (K.A.); 2019207057@njau.edu.cn (Z.W.); 2017207011@njau.edu.cn (X.T.); 2017207046@njau.edu.cn (S.A.L.); 2018207018@njau.edu.cn (Y.Z.); 2017207047@njau.edu.cn (M.A.-u.-H.N.); 2018207024@njau.edu.cn (M.L.); songxiaokai@njau.edu.cn (X.S.); xulixin@njau.edu.cn (L.X.); lixiangrui@njau.edu.cn (X.L.)

**Keywords:** *Haemonchus contortus*, Ephrin domain containing protein, PBMCs, immunomodulation, Th9 immunity

## Abstract

**Simple Summary:**

*Haemonchus contortus* excretory/secretory products (HcESPs) contain many proteins that can perform various functions including modulating the host immune response. Recent studies indicate that IL-9 can be secreted by a specialized population of T cells called Th9 cells, which mediate anti-parasite immunity. Furthermore, HcESPs could enhance goat peripheral blood mononuclear cells (PBMCs) derived Th9 cells production. Ephrin domain containing protein (EPH) was identified as one of the HcESPs that can be isolated from different stages of this helminth. Nonetheless, the understanding of immunomodulatory roles of EPH on Th9 and other immune cells remains limited. In this study, the correlation between recombinant *H. contortus* Ephrin domain containing protein(rHcEPH)and goat PBMCs significantly enhanced Th9 cells differentiation, IL-9 expression, cell apoptosis efficiency, and cell migration, whereas cell proliferation was suppressed significantly depending on the concentration. Our findings illustrated that rHcEPH protein is linked to modulate the host immune cells and could enhance protective immunity by inducing Th9 cells secreted IL-9 in vitro.

**Abstract:**

Ephrin domain containing protein (EPH), a significant excreted and secreted product (ESPs) of *Haemonchus contortus*, has been identified to have antigenic functions. Over the past years, a new subset of CD4 + T named as T helper 9 cells that secrete interleukin-9 (IL-9) as a signature cytokine is associated with tumor immunity and allergy. Nonetheless, the understanding of immunomodulatory roles of EPH on goat Th9 and other immune cells remains limited. Herein, EPH from *H. contortus* (HcEPH) was cloned and expressed in pET-28a. Immunofluorescence assay (IFA) was carried-out to localize rHcEPH within *H. contortus* adult worms and to bind with goat peripheral blood mononuclear cells (PBMCs). Besides, the impact of rHcEPH on signature cytokine IL-9 expression in goat PBMCs was evaluated. Flow cytometry was employed to examine Th9 cells production and cell apoptosis. The results revealed success in the expression and localization of rHcEPH in surface of adult *H. contortus* gut sections. According to IFA analysis, the rHcEPH protein was capable to react precisely with anti-*H. contortus* antibodies. Further functional analysis showed that correlation between rHcEPH and host PBMCs significantly enhanced Th9 cell differentiation, IL-9 expression, cell apoptosis efficiency, and cell migration, whereas cell proliferation was suppressed significantly depending on the concentration. Our observations indicated that rHcEPH protein is linked to modulate the host immune cells and could enhance protective immunity by inducing Th9 responses.

## 1. Introduction

*Haemonchosis* is a parasitic disease caused by gastrointestinal nematode *Haemonchus contortus* [1]. Goats and sheep can become infected upon ingestion of infective larvae which may result in anemia, diarrhea, decrease in weight, and death in some cases [2,3]. The immunomodulatory capacity of *H. contortus*, mediated by parasite-derived effector molecules, is believed to play important roles in the establishment of long-lasting infection in the host. Previously, several attempts were made for the recognition of immunogenic molecules derived from ESPs with potential for vaccine use, such as transthyretin domain- containing protein [1], 14-3-3 Isoform 2 [4], and glyceraldehyde-3-phosphate dehydrogenase [5]. Nematodes are known to release ESPs which can enter and survive inside a host by modulating the host immune responses [6]. Furthermore, ESPs from *H. contortus* has the ability to increase protective immunity by activating Th9 immune cells in vitro [7]. It was demonstrated previously that Ephrin domain-containing protein (EPH) from HcESPs from this parasite in various developmental stages [8]. Moreover, Ephrin plays important roles during anti-cancer therapies, hematopoiesis and proliferation of cells [9,10,11]. However, the exact biological function and immunological characters of HcEPH are not clear.

Peripheral blood mononuclear cells (PBMCs) are comprised of numerous immune cells, which play essential roles in inherent and adaptable immune reactions. After interacting with different antigen, the naïve CD4 + T helper (Th) cells regulate the adaptive immune system by producing specific cytokines [7,12]. Recent studies illuminated that IL-9 can be secreted by a specialized population of T cells called Th9 cells, which mediate anti-parasite immunity by producing IL-9 [13,14,15]. IL-9 cytokine plays an important role in the control of parasite (*Trichinella spiralis*, *Trichuris muris*, and *Nippostrongylus brasiliensis*) infection and pathology [14,16,17,18]. Th9 cells can produce IL-10 while producing IL-9, but not IL-4, IL-5, IL-13 and other cytokines, which can distinguish Th9 cells from other IL-9 producing cells [19]. In our previous study, we evaluated the immunomodulatory effects of HcESPs on the differentiation of Th9 cells and IL-9 cytokine production in vitro, which would help to understand the relationship between host and parasite [7].

In this research, the EPH gene was successfully cloned and recombinant protein was expressed. In addition, the HcEPH in adult *H. contortus* worm was localized. Moreover, potential immune modulatory roles of recombinant HcEPH on goat PBMCs were evaluated in vitro. In addition, a novel role of rHcEPH with detailed impact on how Th9 cells and IL-9 cytokine can be produced in host cells was investigated.

## 2. Materials and Methods

### 2.1. Animals and Parasites

Since specific pathogen free (SPF) goats were not available, four local crossbred female goats (Age, 4–6 months) were obtained from local market of Jiangsu province and placed in animal house (two goats per pen) of Nanjing Agricultural University, they were subject to deworming by levamisole (8 mg/kg body weight) for helminths to be eradicated every 2 weeks apart. A 4-week fecal examination was conducted for the confirmation of helminths free.

Two female Wistar rats (body weight ~200 g) were purchased from Yangzhou University. They were confined to a disinfected space (two rats per cage) in Nanjing Agricultural University and given sterilized food and water.

*H. contortus* third-stage (L3) larvae were obtained from the Laboratory of Veterinary Parasitic Diseases of Nanjing Agricultural University.

Treating animals in the study were in compliance with the guidance issued by the Animal Ethics Committee, Nanjing Agricultural University, China (Approval ID: PZ2019013).

### 2.2. Cloning and Sequence Analysis of H. Contortus EPH Gene

Goats were orally infected with 8000 infective stage larvae (L3) of *H*. *contortus*. Animals were sacrificed following the proper procedure and by national humane euthanasia guidelines at 4 weeks post-infection. After slaughter, the abomasum of each animal was removed and dissected. From each abomasum, adult *H. contortus* worms (mixture of females and males) were picked and washed several times with PBS. According to prior description, Trizol (Invitrogen, Shanghai, China) method was applied to separate RNA [20], prior to cDNA synthesis conducted in line with the requirements set out by the cDNA synthesis Kit supplier (TaKaRa Biotechnology, Sanghai, China). Then, for down-stream applications, it was put in storage at −30 °C.

Based on the sequence of *H. contortus* EPH gene (GenBank: CDJ87334.1), reverse transcriptase PCR (RT-PCR) was performed to amplify the open-reading frame of HcEPH. *SacI* and *HindIII* restriction enzymes were incorporated into the 5′-end of sense primer (5′-TTC GAG CTC ATG ATA CTT CAC ATA TTC TGC-3′) and antisense primer (5′-GAC AAG CTT TTA CAT CCA CAG AAG TGC CAA-3′) for replication. Total 50 μL PCR product was made up of 25 μL Green Taq Mix (Vazyme Biotech, Nanjing, Jiangsu, China), 2 μL of cDNA, 19 μL ddH_2_O and 2 μL of each primer. PCR amplification was performed as follows: Initial denaturing (1 cycle) 95 °C for 180 s, followed by 30 cycles of 95 °C for 15 s, 60 °C for 15 s and 72 °C for 60 s, finally extension at 72 °C for 300 s. One percent agarose gel electrophoresis and an E. Z.N.A. Gel Extraction Kit (Omega bio-tech, Norcross, GA, USA) were used for the identification and purification of amplified fragment of PCR. The obtained product was attached to pMD-19T vector (TaKaRa Biotechnology, Sanghai, China). The recombinant plasmid (HcEPH) was converted into *Escherichia coli* (*E. coli*) strain, DH5α-competent cells, and cultured in Luria Bertini (LB) medium (Tryptone 10 g/L, Yeast extract 5 g/L, NaCl 10 g/L) with ampicillin (100 µg/mL) until optimal density of the culture reached at OD_600_. Recombinant plasmid was purified according to the protocol of plasmid purification kit (Qiagen). The double restriction enzymes *SacI* and *HindIII* were applied to digest the recognizable recombinant plasmid pMD-19T/HcEPH. Subsequently, the HcEPH gene was subject to purification and ligation into the pET-28a (+) prokaryotic expression vector as per instruction of T4 DNA ligation kit (Vazyme Biotech, Nanjing, Jiangsu, China, presented in Instruction S1). To confirm the accurate insertion of HcEPH gene to the framework of pET-28a, the sequencing of recombinant plasmid was conducted.

### 2.3. Expression of Recombined Proteins

The recombinant plasmid (pET-28a(+)/HcEPH) was transformed into *E. coli* BL21 and cultured in Luria Bertini (LB) medium with ampicillin (100 μg/mL) at 37 °C. Before the optic density at 600 nm (OD_600_) hit 0.6~0.8 and recombinant protein was stimulated using 1 mM isopropyl β-D-1-thiogalactopyranoside (IPTG; Sigma Aldrich, Shanghai, China) for 5 h, the positive colonies developed at 37 °C under continued stirring. Afterwards, the obtained cultures were subject to centrifuge at 10,000× *g* for 10 min. Prior to sonication, lysozyme was employed for pellet to be lysed (10 µg/mL, Sigma Aldrich, Shanghai, China). Twelve percent (*w*/*v*) SDS-PAGE was employed to analyze the sonicated substance of the recombined proteins. In line with the guidance from the supplier, Ni2+ nitrilotriacetic acid column was applied to derive purified protein from bacteria lysis (GE Healthcare, Pittsburgh, PA, USA). Finally, SDS-PAGE was applied to conduct analysis of the production of the recombinant proteins and the concentration was obtained by Bradford [21] method. The lipopolysaccharide (LPS) was detoxified from the rHcEPH protein using ToxinEraserTM Endotoxin Removal Kit (GenScript, Nanjing, China). The concentration of the rHcEPH protein was adjusted to 1 mg/mL. The endotoxin level of the rHcEPH protein was determined by the ToxinSensor^TM^ Chromogenic Limulus Amebocyte Lysate (LAL) Endotoxin Assay Kit (GenScript, Nanjing, China) below 0.1 EU/mL.

### 2.4. Western Blot Analysis

With 200 µg of rHcEPH protein blended with Freund’s complete adjuvant (1:1, Sigma Aldrich, Shanghai, China) injected subcutaneously in Wistar rats for the generation of polyclonal antibodies against recombinant protein. After 14 days of initial dose, rats were injected two times (intervals of one week) with the same dose of the rHcEPH protein mixed with incomplete Freund’s adjuvant (1:1, Sigma Aldrich, Shanghai, China). After 10 days of last injection, the blood samples were obtained, and sera sampling were put in storage for later use.

Prior to transferal to nitrocellulose filter membrane (NC, Merck-Millipore, Boston, MA, USA), the rHcEPH was purified and isolated on SDS-PAGE. After blockage with 5% skim milk (BD Biosciences, San Josè, CA, USA) in TBS-T (TBS with 0.05% Tween 20) for 60 min at 37 °C, the membrane was detected with rat-anti-HcEPH antiserum/normal rat serum with a 1:1000 dilution using TBS-T for 60 min at 37 °C. Prior to the incubation with goat anti-rat IgG antibody connected to horseradish peroxidase (HRP) (Thermo Fischer Scientific, Waltham, MA, USA) with a 1:5000 ratio in blockage buffer for 45 min at 37 °C, the membrane was subject to washing for 5 times (5 min each) with TBS-T. With 3,3-diaminobenzidine tetrahydrocholoride (DAB, Tiangen Biotech, Beijing, China) substrate used, the protein band was confirmed as the chromogenic substrate.

### 2.5. Localization Assay

Before being frozen quickly in liquid nitrogen, the *H. contortus* adult worms were subject to suspension in TISSUE-TEK^®^ O.C.T. compound (SAKURA, Torrance, CA, USA). The worms were cut into 10-µm thickness sections by cryotome (CM1950, Wetzlar, Germany), before being fixed (4% Paraformaldehyde) using poly-l-lysine hydrobromide glass slides. The slides were treated with five percent bovine serum albumin (BSA, Sunshine Bio, Nanjing, Jiangsu, China) to block non-specific bindings followed by incubation with 1:1000 dilutions of rat-anti-EPH serum (experimental group) or normal rat serum (control group) as primary antibody and Cy3-labeled Goat Anti-Rat IgG (1:500 dilutions) as secondary antibody (Beyotime, Shanghai, China), incubation was conducted at 37 °C for 50 min. To facilitate nuclei coloring, the parts were labeled using 1 mg/mL of 4′,6-diamidino-2-phenylindole (DAPI: Sigma, St Louis, MO, USA) for 5 min. Finally, the samples were mounted in ProLong Gold antifade reagent (Beyotime, Shanghai, China) and evaluated by a confocal laser-scanning microscope.

### 2.6. Preparation of Goat PBMCs

According to previous study, jugular vein of dewormed donor female goats (uninfected; helminths free) was taken as the source of blood samples and gradient centrifugation technique was applied to separate PBMCs [22,23]. Cell density was adjusted to 1 × 10^6^ cell/mL in RPMI 1640 containing 10 percent heat-inactivated FBS (Fetal bovine serum), 100 U/mL penicillin after washing twice with Ca^2+^/Mg^2+^-free PBS (pH 7.4). For cell viability to be assessed, Trypan blue exclusion test was conducted.

### 2.7. PBMCs Binding Assay

According to prior description, an immunofluorescence assay (IFA) was conducted to validate interaction [24]. In brief, 10 μg/mL rHcEPH (experimental group) or phosphate-buffered saline (PBS, control group) were used separately for a 2-h incubation of the freshly isolated PBMCs (1 × 10^6^ cells/mL) in a humidified atmosphere with 5 percent CO_2_ at 37 °C. After washing, cells were allowed to rest on poly-L-lysine treated glass pieces for 15 min prior to being fixated in four percent paraformaldehyde for a quarter of hour at room temperature. Afterwards, the cells were subjected to the blockage agent (5% BSA in PBS) at 37 °C for 1 h to reduce backdrop coloring to the minimum. Cell incubation was conducted using rat anti-rHcEPH sera (1:100 dilutions; primary antibody) for 1 h. Before incubating Cy3-labeled Goat Anti-Rat IgG (1:500 dilutions; secondary antibody; Beyotime, Shanghai, China) for 45 min. The cells underwent counterstaining with DAPI (Sigma, MO, USA) for 5 min. Stained cells were imaged using a confocal laser scanning microscope (Nikon, Tokyo, Japan).

### 2.8. Cell Activity Assay 

Cell activity index was tested by cell counting kit-8 (CCK-8) reagents (Beyotime Biotechnology, Jiangsu, China) according to the protocol described previously [23]. In brief, isolated goat PBMCs (1 × 10^6^ cells/mL) were washed with Ca^2+^/Mg^2+^-free PBS (pH 7.4). One hundred microliters of cells containing RPMI 1640 + FBS/antibiotic medium were poured in 96-well plate with different concentrations of rHcEPH (10, 20, and 40 μg/mL), PBS (Negative control) or ConA (10 μg/mL, Positive control) at 37 °C with 5 percent CO_2_ for 72 h. Ten microliters of the CCK-8 reagent was applied in each well for 4 h and then absorbance value (OD450) was read in micro plate reader (Thermo Scientific, Minneapolis, MN, USA).

### 2.9. Cell Migration Assay

To facilitate the transfer of PBMCs through polycarbonate membrane with 8 µm pores, the Trans-well system was applied to perform migration activity in triplicate (Merck-Millipore, Boston, MA, USA) [25]. Isolated goat PBMCs (1 × 10^6^ cells/mL) were washed with Ca^2+^/Mg^2+^-free PBS (pH 7.4). Cells containing RPMI 1640 + FBS/antibiotic medium were scattered into a 24-well plate (1 mL/well) with different concentrations of rHcEPH (10, 20 and 40 μg/mL), PBS (control) at 37 °C with 5 percent CO_2_. Result were analyzed in the proportion of seeded PBMCs.

### 2.10. Annexin V-FITC/Propidium Iodide Staining Assay

Freshly isolated goat PBMCs (2 × 10^6^ cells/mL) were washed with Ca^2+^/Mg^2+^-free PBS (pH 7.4). Cells containing RPMI 1640+FBS/antibiotic medium were added into 24-well plate (1 mL/well) with varying concentrations of rHcEPH (10, 20, and 40 μg/mL) and PBS (control) and incubated at 37 °C in a humidified atmosphere with 5 percent CO_2_ for 15 h. The PBMCs were centrifuged at 300× *g* for 10 min before washing thrice by PBS. Then, the PBMCs were suspended in the 1× buffer, while the cells were incubated in the dark place using annexin V-FITC (Miltenyi Biotec, Bergisch Gladbach, Nordrhein-Westfalen, Germany) and propidium iodide (Miltenyi Biotec, Bergisch Gladbach, Nordrhein-Westfalen, Germany) for a quarter of an hour at room temperature. The efficiency of rHcEPH in inducing PBMCs apoptosis was obtained by flow cytometry (FACS, BD Biosciences, San Josè, CA, USA) analysis.

### 2.11. Detection of Cytokine Transcripts by FQ-PCR

Fluorescence-quantitative real time PCR (FQ-PCR) was used to determine the expression of IL-9. Initially, the goat PBMCs were isolated and incubated using varying concentrations of rHcEPH (10, 20, and 40 μg/mL) and PBS (control). In accordance with to the guidance of manufacturer, TRIzol (Invitrogen, Carlsbad, CA, USA) was applied to extract total RNA and cDNA kit was employed to conduct cDNA synthesis (Vazyme Biotech, Nanjing, Jiangsu, China). To ascertain the expression of IL-9, FQ-PCR was employed. The *β*-actin gene was used as reference gene. According to our previous study [7], primer sequences presented in Supplementary Materials Table S1 were used in this study.

### 2.12. Determination of Goat PBMCs-Derived T helper-9 Cells

As referred to before, flow cytometry was employed to ascertain how markers are expressed on T cells from PBMCs [12,26,27]. Varying concentrations of rHcEPH (10, 20, and 40 μg/mL) and PBS (control) were used to culture the goat PBMCs for 48 h. Then, at 37 °C in a humidified atmosphere with 5 percent CO_2_, the incubation of phorbol myristate acetate (50 ng/mL; Sigma, St Louis, MO, USA) and ionomycin (1 μg/mL; Sigma, St Louis, MO, USA) was carried out [7]. Brefeldin A (10 μg/mL; Sigma, St Louis, MO, USA) was mixed after 3 h of stimulation, and cells were obtained and washed using PBS after 6 h of stimulation. Subsequently, cells stained with anti-goat surface monoclonal antibodies conjugated with FITC (CD2; Clone-CC42) and Alexa Flour 647 (CD4; Clone-44.38) for 30 min at 4 °C. Afterwards, cells were centrifuged at 500 rcf for 5 min at 4 °C, added 500 μL of Fixation and permeabilization solution (BD Pharmingen, San Jose, CA, USA) for 20 min at 4 °C. Consequently, cells were stained with anti-goat intracellular cytokine monoclonal antibodies, IL-9 (PE-cy5; BD Pharmingen) and IL-10 (PE; BD Pharmingen) in addition to evaluate the expression of Th9 cells. Finally, flow cytometry was performed (setting the gate on CD2 + CD4 + T cell) by using intracellular antibodies (IL-9 and IL-10) to count the Th9 cells, acquiring the gate at 1000, on FACS Canto II flow cytometer (Becton Dickinson). 

### 2.13. Statistical Analysis

Statistical analyses were performed using GraphPad Prism 7.0 (GraphPad Prism, San Diego, CA, USA) software. All data obtained from the above experiments were displayed as mean ± SEM (standard error of the mean). One-way ANOVA followed by Tukey’s post-hoc test was employed to compare the variances between groups and considered statistically significant at * *p* < 0.05, ** *p* < 0.01, *** *p* < 0.001, **** *p* < 0.0001. Flow cytometry data were analyzed using Flow Jo (Version 9) software.

## 3. Results

### 3.1. Molecular Cloning, Expression, Purification, and Western Blot Anaylsis of HcEPH

**Figure 1:**
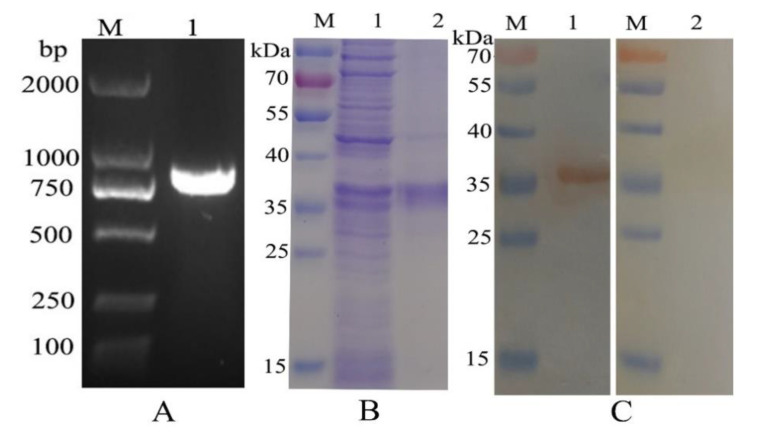
(**A**): PCR product of *Haemonchus contortus* Ephrin domain containing protein (HcEPH) gene; (**B**): expression and purification of rHcEPH, Lane 1: Before purification of rHcEPH, Lane 2: Purified rHcEPH. (**C**): Western blot analysis of rHcEPH. Lane M: Standard protein molecular weight marker; Lane 1: rHcEPH protein detected by anti-rHcEPH rat sera; Lane 2: rHcEPH protein was not detected by normal rat sera.

The fragments of HcEPH genes (792 bp) cloned into a pET-28a (expression vectors) were substantiated by sequence analysis (Figure 1A). This showed that HcEPH was inserted into the vectors with success. Subsequently, the expression and purification of recombinant protein of HcEPH were conducted. Displayed on SDS-PAGE, the purified recombinant protein possessed a molecular mass of roughly 36 kDa (Figure 1B, HcEPH, 29.8 ligated with pET28a, 6.2). The rat-anti-rHcEPH serum is capable of recognizing recombinant HcEPH as shown in immuno-blot analysis. Nevertheless, normal rats’ sera failed to indicate the existence of protein (Figure 1C).

### 3.2. HcEPH Localized of H. contortus Adult Worm Sections

**Figure 2:**
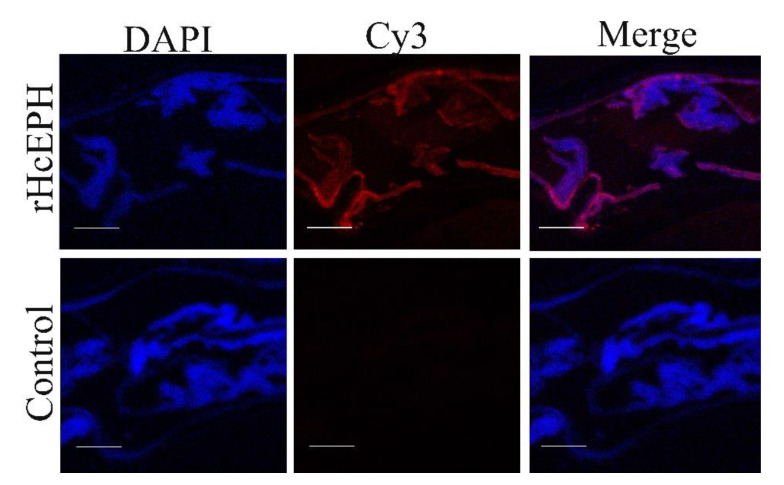
Localization of HcEPH within adult *H. contortus* by immunofluorescence assay. The red color indicates the localization of target protein stained with Cy3 in adult worms and blue color indicate the localization of nuclei stained with DAPI. Merge image of DAPI (blue color) and Cy3 (red color). No red fluorescence was observed in control. Scale-bars 40 µm.

Presence of native HcEPH was observed in *H. contortus* adult worms (Figure 2). Immunohistochemical analysis was performed for ascertaining how native HcEPH is localized inside the *H. contortus* adult worms. The worms were incubated with primary antibodies rat-anti-EPH serum (experimental group) or normal rat serum (control group)], maintained with Cy3-labeled Goat Anti-Rat IgG (secondary antibody) and subsequently counter stained with DAPI. Results indicated that worms incubated with rat-anti-EPH serum showed localization of HcEPH (red color) in the outer and inner surface of membrane as well as in the gut region of parasite. However, no protein localization was detected in worms incubated with normal rat serum (control) and Cy3-labeled Goat Anti-Rat IgG secondary antibody.

### 3.3. Validation of rHcEPH Binding with PBMCs

**Figure 3:**
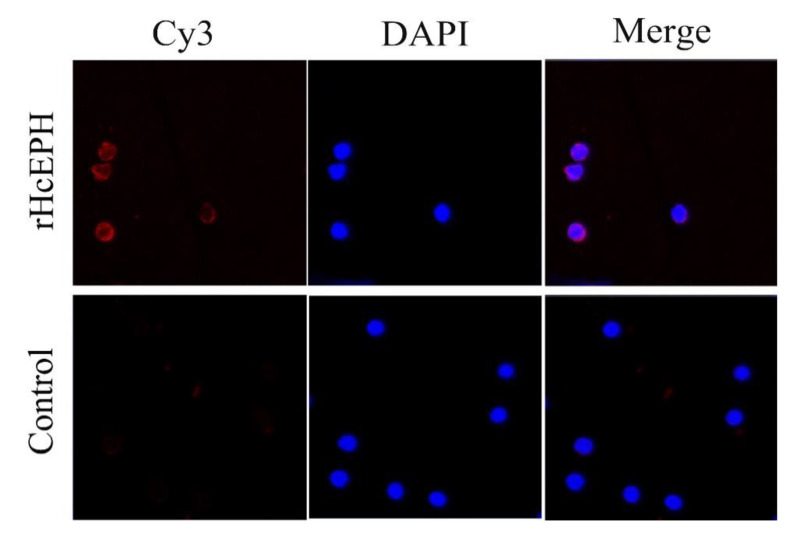
rHcEPH protein binding to goat peripheral blood mononuclear cells (PBMCs) in vitro by immunofluorescence assay. Binding was performed by incubating PBMCs treated with purified rHcEPH protein or sham-treated with PBS (control). Red fluorescence on surface of cells showed target protein staining (Cy3-conjugated secondary antibody) and nuclei of cells were visualized by DAPI (blue), Merge combination of red and blue channels. No protein (red fluorescence) binding was observed in control group. Scale bar 40 µm.

Figure 3 illustrates how rHcEPH binds to the surface of PBMCs isolated from blood of uninfected goats. By incubating rHcEPH-treated PBMCs with specific anti-rHcEPH antibodies, we detected an even distribution and localization of the red Cy3 dye on the PBMCs membrane, indicating the successful binding of rHcEPH protein to PBMCs surface. No red fluorescence was observed in PBS-treated goat PBMCs (control) when incubated with Cy3-labeled Goat Anti-Rat IgG secondary antibody.

### 3.4. rHcEPH Reduced Cell Activity of Goat PMBCs

**Figure 4:**
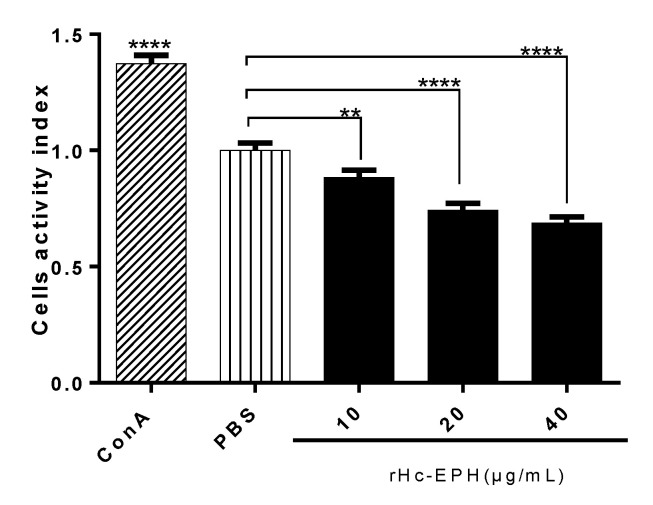
Effects of rHcEPH on PBMCs activity. PBMCs were treated with serial concentrations of rHcEPH, PBS (Negative control) and ConA (Positive control) for 72 h. Cell activity index was calculated with the OD_450_ values in PBS control considered as 100%. The data is expressed as mean ± SEM of three independent experiments (** *p* < 0.01, **** *p* < 0.0001).

CCK-8 reagent kit was employed to assess the activity effects of rHcEPH on host cells. According to the comparative assessment of the cells stimulated by rHcEPH protein, relative to the PBS group, the activity of cells was notably reduced depending on dosage (Figure 4).

### 3.5. rHcEPH Increased Cell Migration of Goat PMBCs

**Figure 5:**
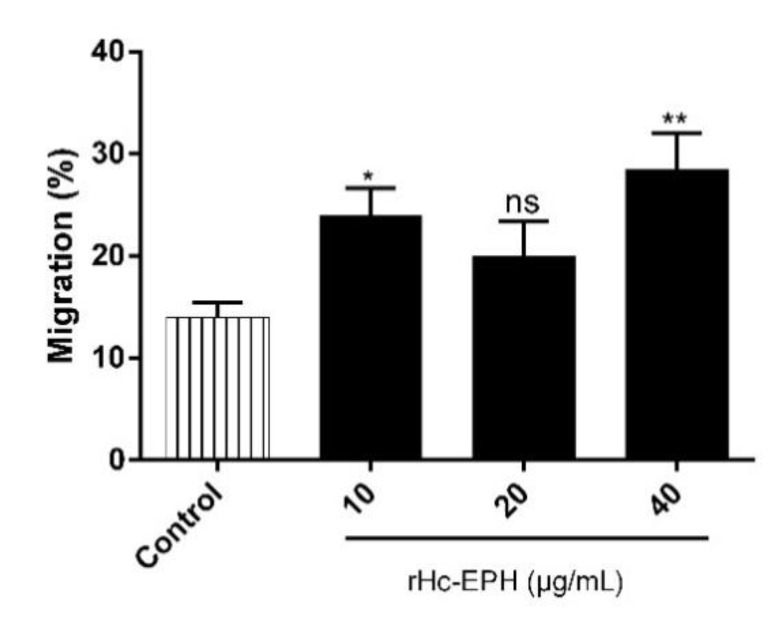
Effects of rHcEPH on PBMCs migration. PBMCs were treated with serial concentrations of rHcEPH and PBS. The migration percentage was determined randomly, and the data is denoted as mean ± SEM of three independent experiments (* *p* < 0.05, ** *p* < 0.01 and ns: no significant difference).

In order to explore the impact of the rHcEPH on cell migration, a Transwell system was applied to carry out cell migration assay (Figure 5). Results showed that cells of the control group were migrated into lower chamber by 14 ± 1%. At concentrations of 10 μg/mL (24.00 ± 1.528%) and 40 μg/mL (28.50 ± 2.500%) of rHcEPH protein, large numbers of cells were migrated through membrane. Among 20 μg/mL (20.00 ± 2.000%) protein concentration and PBS groups; however, only insignificant effects were noted.

### 3.6. rHcEPH Dramatically Modulated Apoptosis of Goat PBMCs

**Figure 6:**
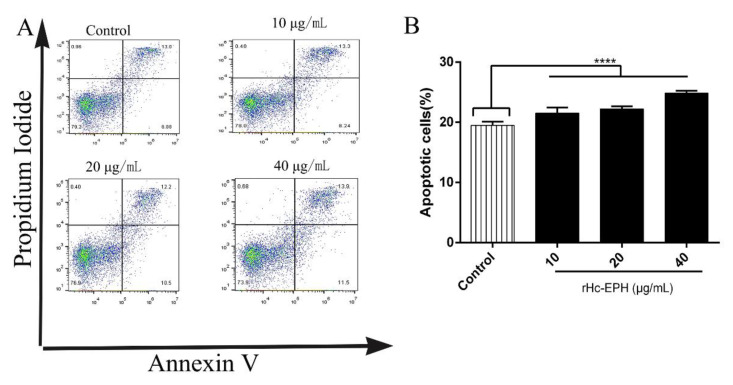
Measurement of rHcEPH on PBMCs apoptosis by flow cytometry. PBMCs were treated with serial concentrations of rHcEPH and PBS. (**A**): Dot plots showing apoptosis (early and late stage) of PBMCs in response to exposure to rHcEPH protein, (**B**): Percentage of apoptosis (early and late stage) of PBMCs after rHcEPH treatment. The data is denoted as mean ± SEM of three independent experiments (**** *p* < 0.0001).

The Annexin V-FITC/PI double coloring apoptosis analysis was conducted to assess the effect of apoptosis in rHcEPH-treated PBMCs. Flow cytometry analysis indicated that cells treated with different rHcEPH concentrations (10, 20 and 40 μg/mL) exhibited significantly increased cell apoptosis when compared with the control (ANOVA: F (3, 20) = 77.10, *p* < 0.0001) (Figure 6).

### 3.7. rHcEPH Modulated IL-9 Secretion by PBMCs

**Figure 7:**
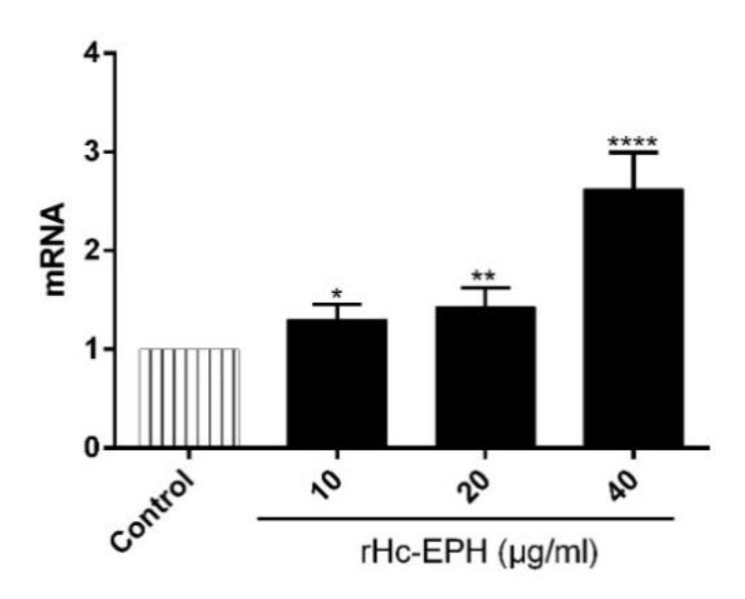
Relative expression of interleukin-9 (IL-9) cytokines in goat PBMCs stimulated by rHcEPH was evaluated. Cells were incubated with serial concentrations of rHcEPH and PBS for 12 h, the mRNAs were quantified by FQ-PCR. The data is expressed as mean ± SEM of three independent experiments (* *p* < 0.05, ** *p* < 0.01, **** *p* < 0.0001).

In order to evaluate the IL-9 production by PBMCs subject to stimulation with serial concentrations of rHcEPH, FQ-PCR assay was conducted. As shown in Figure 7, transcription level of IL-9 (ANOVA: F (3, 14) = 63.64, *p* < 0.0001) was improved significantly at dose-dependent manner when compared to that of control group.

### 3.8. Effect of rHcEPH on Th9 Cells Differentiation

**Figure 8:**
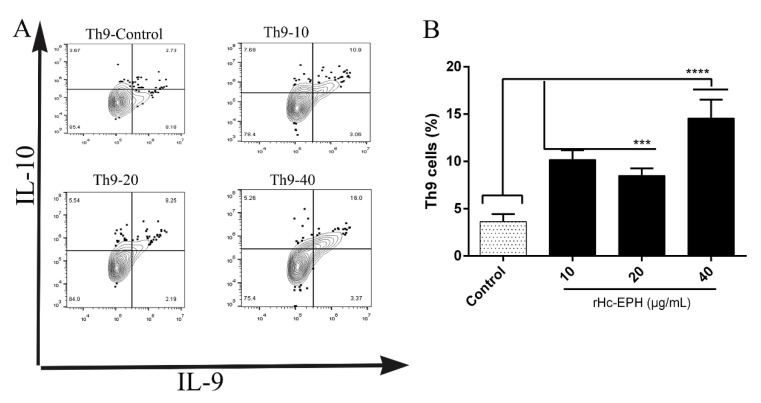
Measurement of rHcEPH on Th9 cells differentiation by flow cytometry. The plots shown are gated on CD2 + CD4 + T cells and using representative intracellular cytokine antibodies (IL-9 and IL-10). (**A**): Contour plots analysis and proportion of PBMCs derived-Th9 cells treated with serial concentrations of rHcEPH and PBS; (**B**): Percentage of differentiation of Th9 cells after rHcEPH treatment. The data is denoted as mean ± SEM of triplicate independent experiments (*** *p* < 0.001, **** *p* < 0.0001).

To further assess the impact of rHcEPH on Th9 cells, serial concentrations of rHcEPH (10, 20, and 40 μg/mL) were cultured with goat PBMCs and stained with CD2 + CD4 + T cell markers. For Th9 cell calculation, the markers for IL-9 and IL-10 were applied and evaluated by flow cytometry. A potential capability was displayed by the rHcEPH regarding the induction of IL-9 cytokine expression. The findings on differentiation of Th9 cells in response to rHcEPH revealed that, relative to the control groups, a significant induction of Th9 cells production was contributed by rHcEPH protein at 10, 20, and 40 μg/mL. (Figure 8).

## 4. Discussion

Anthelmintic drugs remain the mainstay of *H. contortus* control, which is becoming difficult because of the resistance developed by parasites to these medications. Therefore, prophylaxis against gastrointestinal nematode infections by vaccine would possibly be a successful approach to protect animals. An earlier study has indicated that immunization with HcESPs could protect sheep against nematode infection [28]. Previously, proteins of Hcftt-2, Hc24, and Hc40 from the HcESPs have proven to be the promising candidates for haemonchosis vaccine [1,4,20]. Moreover, a vaccine (Barbervax^®^, WormVax, Moredun Research Institute, Scotland, UK) containing two native integral gut membrane proteins from *H. contortus* (H11 and H-gal-GP), was licensed for commercially use in Australia [29]. However, geographic spread restrictions and required repeated vaccination to stimulate high antigen-specific circulating antibody levels have limited its reach to the global market [30]. Therefore, development of an effective, safe and durable vaccine against *H. contortus* infection would possibly be a successful approach to protect animals. In this study, we characterized EPH, one of the ESPs proteins, and evaluated its immune functionalities in the host immune cells. We showed that rHcEPH after incubation with goat PBMCs could bind to the surfaces of PBMCs as confirmed via IFA. Its absorption by host immune cells, which is shown to be complicated by superficial ions, a striking feature of ESPs substances to regulate the immune functionalities performed by host PBMCs and is consistent with the results of prior research [31]. In our previous in vivo study, liquid chromatography-tandem mass spectrometry (LC-MS/MS) analysis was found EPH at different developmental stages of *H. contortus* [8]. In this study, immunohistochemically HcEPH was localized in adult worms of *H. contortus*. Our results suggested that HcEPH could attach on the surface of cells to regulate immune functions during host–parasite interface.

ESPs molecules from helminths are considered to play important immunoregulatory roles in vivo as well as in vitro during host–parasite interaction [8]. In immunological processes, both antigen-presenting cells and T cells are crucial for the proliferation of immune cells [32]. It has been reported that migration and proliferation of host immune cells are crucial for the control of parasite infection [33]. Herein, PBMCs activity was decreased at significant level, which ultimately increased the percentage of cell apoptosis in response to rHcEPH treatment. Our study indicated that rHcEPH as a constituent of HcESPs might interfere with modulatory activity on PBMCs proliferation and cell death regulation; however, the actual mechanism and pathways involved in the adjustment of this apoptosis and proliferation need to be studied further. The cellular mechanisms including cell migration play a significant role in immune surveillance by attracting immune cells to kill infectious pathogens and promote regeneration of surrounding tissues. In addition, it was demonstrated that parasites dynamically induce migration of host immune cells at the site of infection [34]. In this study, it was showed that rHcEPH protein increased the migration rate of immune effector cells. These results are in line with our previous work [12]. Additionally, further studies are needed to confirm the actual mechanism of cell migration.

IL-9 is a cytokine classically associated with Th2 type immune response. However, its cellular identification has recently been reassessed to recognize a new specialized T helper subset called Th9 [35]. Previously, Th9 has been related to tumor immunology, allergic responses and autoimmunity; until now, various roles of Th9 cells have been reported in animal models during gastrointestinal parasites infection [7,36,37,38]. In our recent study, it was demonstrated that interaction of HcESPs with PBMCs enhanced the Th9 cells production and expression of IL-9 cytokine [7]. From a functional perspective, IL-9 was discovered to induce innate and adaptive immune responses [39]. Helminth infection’s faster expulsion from gastrointestinal tract is enhanced by IL-9 [40,41]. Thus, Th9 cells produce IL-9 which has been shown to be clearly efficient in driving basophilia, increased mast cell numbers and led to rapid parasitic removal [18]. Herein, PBMCs stimulated with serial treatments of rHcEPH promoted Th9 amounts and expression of IL-9 depending on dosage in vitro. To our knowledge, this is the first study on the effects of Ephrin domain containing protein on Th9 immune response during *H. contortus* infection. However, it is necessary for further studies to be conducted for exploring the underlying mechanisms of rHcEPH impacting Th9 immunity in various phases in goat.

Recombinant protein vaccines offer a more attractive option for commercially viable vaccines. In this study, indicated that recombinant HcEPH is important active protein of *H. contortus* ESPs that played crucial roles in the immune regulations. Similar to *H contortus*, a series of candidate vaccine antigens for sheep abomasal parasite *Teladorsagia circumcincta* have been screened over the years. The native versions of these antigens were originally identified either as immunodominant ESPs or selected because of their potential Immunomodulatory role [42]. Additionally, vaccination with a fraction of *Ostertagia ostertagi* adult ESPs has significantly reduced worm egg output after infections [43]. However, Further studies are needed to understand whether rHcEPH can be used as a candidate vaccine antigen.

## 5. Conclusions

In the current observations, it was highlighted that, as an important and active constituent of HcESPs localized in adult worms, rHcEPH plays a crucial role in the cellular immune regulations. According to the results, rHcEPH could bind with host immune cells, enhanced migration of goat PBMCs, IL-9 expression, Th9 cell generation, as well as cell apoptosis. However, cell activity was decreased in a dose dependent manner. Collectively, rHcEPH could contribute to the induction of Th9 immune response in goats during *H. contortus* infection, which would help to elucidate the host–parasite interaction. Our data provide a detailed understanding of the immune functionalities of rHcEPH in goat PBMCs that could be helpful for the future studies aimed at therapeutic strategies against Haemonchosis.

## Supplementary Materials

The following are available online at https://www.mdpi.com/2076-2615/10/11/2137/s1, Table S1: Primer sequences for FQ-PCR, Instruction S1: Instructions of T4 DNA ligation kit.
