# Supplementary material for: Unveiling the Immunomodulatory Characteristics of Haemonchus contortus Ephrin Domain Containing Protein in the Parasite–Host Interactions
Source: Animals (Basel). 2020 Nov 17;10(11):2137. doi: 10.3390/ani10112137 (PMC7698521; doi:10.3390/ani10112137)
Supplement: Supplementary file 1 [file animals-10-02137-s001.zip › animals-943199 - supplementary Instruction.pdf]

Introduction

The T4 DNA Ligase catalyzes the formation of a phosphodiester bond between the adjacent 5'-phosphate and 3'-hydroxyl on the blunt or cohesive end of dsDNA. It can also catalyze the linkage of RNA with ssDNA or RNA in double stranded nucleic acids. However, it cannot catalyze linkages between single stranded nucleotides. The T4 DNA Ligase can be used in labelling the 3'-end of RNA, cyclizing RNA and DNA oligonucleotides, cloning of cDNA, and other manipulation of nucleic acids.

Package Information

**Components**	**C301-01 40,000 U**
10× Ligase Buffer*	1 ml
T4 DNA Ligase (400 U/μl)	100 μl

Storage

Store at -20℃.

Unit Definition

In a ligation reaction system of 20 μl, one unit (U) is defined as the amount of enzyme required to catalyze the ligation of more than 50% of 6 μg λDNA-HindIII DNA fragments in 30 min at 16℃.

Application

1. Ligation between DNA fragments and vector DNA.
2. Ligation between DNA fragments and Linker or adaptor DNA.

Protocol

1. Prepare the following reaction solution in a microcentrifuge tube:

10× Ligase Buffer	1 μl
Insert DNA [a]	0.3 pmol
Vector DNA [b]	0.03 pmol
T4 DNA Ligase (400 U/μl)	1 μl
Sterile distilled water	to 10 μl

Note: 1. The molar ratio of Insert/Vector should be between 3: 1 and 10: 1.
2. The blunt-end vector should firstly be dephosphorylated to avoid self-cycling.

2. Incubate overnight at 16℃.

3. Transformation.

3.1. Add the ligation product to 100 μl of competent cells. The volume of the ligation product should be less than 1/6 of the volume of competent cells. Mix gently and incubate for 30 min on ice.

3.2. Incubate the mixture at 42℃ in a water bath for excatly 90 seconds. Then immediatly chill on ice for 2 min-3 min without disturbing the mixture.

3.3. Add 900 μl of LB or SOC medium to the centrifuge tube. Then out the tube in a shaker-incubuator (150 rpm, 37℃) for 45 min, during which the cells will recover and express the resistance gene.

3.4. Centrifuge at 2,500×g for 5 min and discard 900 μl of supernatant. Resuspend the cells with the remaining medium and gently coated on a agar plate containing the appropriate antibiotics. Incubate overnight at 37℃.
