# Supplementary material for: Unveiling the Immunomodulatory Characteristics of Haemonchus contortus Ephrin Domain Containing Protein in the Parasite–Host Interactions
Source: Animals (Basel). 2020 Nov 17;10(11):2137. doi: 10.3390/ani10112137 (PMC7698521; doi:10.3390/ani10112137)
Supplement: Supplementary file 1 [file animals-10-02137-s001.zip › animals-943199 - supplementary Table.pdf]

Table 1. Primer sequences for FQ-PCR.

Gene name	5'–3'	
	**Forward**	**Reverse**
β-actin	CACCACACCTTCTACAAC	TCTGGGTCATCTTCTCAC
IL-9	GATGCGGCTGATTGTTT	CTCGTGCTCACTGTGGAGT
